# Multivariate Models of Performance Validity: The Erdodi Index Captures the Dual Nature of Non-Credible Responding (Continuous and Categorical)

**DOI:** 10.1177/10731911221101910

**Published:** 2022-06-25

**Authors:** Laszlo A. Erdodi

**Affiliations:** 1 University of Windsor, Ontario, Canada

**Keywords:** neuropsychology, performance validity assessment, multivariate models, embedded PVTs, Erdodi Index

## Abstract

This study was designed to examine the classification accuracy of the Erdodi Index (EI-5), a novel method for aggregating validity indicators that takes into account both the *number* and *extent* of performance validity test (PVT) failures. Archival data were collected from a mixed clinical/forensic sample of 452 adults referred for neuropsychological assessment. The classification accuracy of the EI-5 was evaluated against established free-standing PVTs. The EI-5 achieved a good combination of sensitivity (.65) and specificity (.97), correctly classifying 92% of the sample. Its classification accuracy was comparable with that of another free-standing PVT. An *indeterminate* range between *Pass* and *Fail* emerged as a legitimate third outcome of performance validity assessment, indicating that the underlying construct is an inherently continuous variable. Results support the use of the EI model as a practical and psychometrically sound method of aggregating multiple embedded PVTs into a single-number summary of performance validity. Combining free-standing PVTs with the EI-5 resulted in a better separation between credible and non-credible profiles, demonstrating incremental validity. Findings are consistent with recent endorsements of a three-way outcome for PVTs (*Pass*, *Borderline*, and *Fail*).

The clinical interpretation of the results of neuropsychological evaluation rests on the assumption that test scores accurately reflect the examinee’s true ability level (Lezak et al., 2012). However, some test takers are either unwilling or unable to demonstrate their highest or even typical cognitive functioning during psychometric testing. There is an emerging consensus within the field that the credibility of neurocognitive profiles should be evaluated systematically, using multiple objective measures (Bush et al., 2005, 2014; Chafetz et al., 2015; Heilbronner et al., 2009; Lippa, 2018) dispersed throughout the assessment (Boone, 2009).

This mandate often conflicts with growing systemic pressures to abbreviate test batteries in an increasingly cost-conscious health care climate. Expansive, multi-trial free-standing performance validity tests (PVTs) are often the first measures to be sacrificed in response to such external factors (Lichtenstein et al., 2018). Combining evidence from embedded validity indicators (EVIs) provides a more realistic practical solution for building multivariate models of performance validity. EVIs are validity cutoffs developed within existing measures of cognitive ability. In addition to cost-effectiveness (i.e., no extra demands in terms of test material, clinician time, and patient stamina), EVIs have the advantage of inconspicuously monitoring performance validity. Therefore, they are resistant to coaching (Miele et al., 2012; Rohling & Boone, 2007) and protect assessors from the appearance of bias toward malingering detection (Boone, 2013). They also allow assessors to retrospectively evaluate the credibility of neuropsychological profiles that lack free-standing PVTs.

Naturally, EVIs have their own set of limitations: being nested within a test of neuropsychological functioning, they are criticized for conflating invalid performance with genuine impairment (Bigler, 2014) and for their inferior classification accuracy relative to free-standing PVTs (Martin et al., 2015; Nussbaum et al., 2022; Shura et al., 2020). When applied to individual instruments, these objections are justified. However, combining multiple EVIs improves their overall signal detection profile (Erdodi, 2021; Larrabee, 2008). Requiring multiple failures at a liberal cutoff (i.e., one designed to optimize sensitivity over specificity) significantly reduces the multivariate base rate of failure (BR_*Fail*_; Pearson, 2009). In other words, while isolated failures are common (likely signaling an elevated risk for false positives), the BR_*Fail*_ associated with certain *combinations* of EVI failure are quite low (Pearson, 2009), effectively constraining the false positive rate both in theoretical (Berthelson et al., 2013; Medici, 2013; Odland et al., 2015; Silk-Eglit et al., 2015) and empirical (Davis & Millis, 2014; Larrabee, 2014a, 2014b) models. In fact, counting the number of PVT failures (embedded or free-standing) is a widely accepted methodology for establishing criterion groups in research studies (Schroeder et al., 2021) and by extension, determining the credibility of a given profile in clinical and forensic settings.

Depending on the number and quality of PVTs available, failing ≥2 (Dandachi-FitzGerald et al., 2020) or ≥3 (Larrabee et al., 2019) provides strong evidence of invalid performance. Some argued that even a single PVT failure raises doubts about the credibility of a response set (Proto et al., 2014). An apparent limitation of counting the number of failed PVTs is that for each measure, a categorical outcome (*Pass/Fail*) is established along a single cutoff, imposing an artificial dichotomy on performance validity, which is an inherently continuous variable (Erdodi, 2019; Lippa et al., 2014). Several PVTs have multiple cutoffs, ranging from liberal to conservative (i.e., optimized for specificity). Failing the liberal cutoff but passing the conservative one creates uncertainty about clinical interpretation, largely because the predictive value of such “in-between” scores on PVTs is rarely studied systematically. The lack of empirical evidence invites subjective labeling. This indeterminate range has been called both “near-*Pass*” (Bigler, 2012) and “soft *Fail*” (Erdodi & Lichtenstein, 2017). The variability in terminology reflects the inherent ambiguity in the clinical interpretation of such a score.

In practice, the issue around levels of PVT failure is often resolved by simply choosing *one* of the available cutoffs without much reflection on the decision-making process. However, the choice of cutoff does influence the outcome of PVT research and therefore, has important methodological, clinical, and forensic implications. Naturally, BR_*Fail*_ is determined by the cutoffs used. The cumulative effect of using liberal versus conservative cutoff can result in large differences in BR_*Fail*_ (Abeare et al., 2018; An et al., 2017; Pearson, 2009). In addition, the level of cutoff has an invisible, yet potentially significant effect on classification accuracy. Liberal cutoffs run the risk of misclassifying borderline cases as *invalid*, thus diluting the relative concentration of non-credible responding in the *Fail* group. Conversely, conservative cutoffs run the risk of misclassifying borderline cases as *valid*, thus contaminating the *Pass* group with cases of questionable credibility. Either choice will attenuate classification accuracy (Soble et al., 2020).

Erdodi, Roth, et al. (2014) introduced a model for combining several independent validity indicators (one free-standing PVT and six EVIs) into an aggregate measure of performance validity. Although it has been mostly used to combine EVIs only since then, the model is flexible by design and thus, allows the aggregation of any number and type of PVTs (i.e., free-standing or embedded). Instead of relying on a single cutoff, the Erdodi Index (EI) was designed to capture both the *number* and *extent* of PVT failures. As such, the EI model takes into account the cumulative evidence within a multivariate model of performance validity assessment and provides a single-number summary for the credibility of a given neurocognitive profile. The EI model classifies examinees into one of three categories: *Pass*, *Borderline*, and *Fail*. Given that both research designs and clinical decision making require dichotomous outcomes (valid/invalid), the middle category is excluded from criterion grouping. In applied settings, assessors can use clinical judgment in their interpretation of *Borderline* profiles.

In a follow-up study, Erdodi (2019) consolidated earlier arguments for recognizing “indeterminate” (i.e., *Borderline*) as a legitimate third outcome of performance validity assessment, a position that has since been embraced by the American Academy of Clinical Neuropsychology (Guilmette et al., 2020). In a mixed clinical sample of 234 adults referred for neuropsychological assessment, the *Borderline* range was significantly different from both *Pass* (i.e., stronger evidence of non-credible responding) and *Fail* (i.e., weaker evidence of non-credible responding). These findings are consistent with the results of previous (Erdodi & Rai, 2017; Erdodi, Sagar, et al., 2018; Erdodi, Seke, et al., 2017) and subsequent (Abeare, An, et al., 2021; Cutler et al., 2021; Dunn et al., 2021; Erdodi, Hurtubise, et al., 2020) investigations.

The EI model has the potential to become a useful template for aggregating PVTs, both for clinical and research purposes. It is flexible in terms of number and type of component PVTs, allowing practitioners to customize it to meet their unique assessment needs. However, despite a growing body of literature attesting its utility (Erdodi, 2021; Erdodi et al., 2016; Erdodi, Pelletier, & Roth, 2018), the EI has never been calibrated against multiple well-established free-standing PVTs, nor has it been directly compared with another free-standing PVT. This study was designed to address this gap in the literature, and evaluate the classification accuracy of a five-variable version of the EI using three of the Green family of free-standing PVTs as criterion measures. In addition, the classification accuracy of the EI-5 was compared with that of the first trial of the Test of Memory Malingering (TOMM-1).

## Method

### Participants

A consecutive case sequence of 452 patients was selected from an archival database at a private practice from Western Canada. Patients were assessed between 2006 and 2014. The majority of the sample was male (54.6%) and right-handed (90.7%). Inclusion criteria were age between 18 and 69 and a full administration of the following tests: Word Memory Test (WMT), Medical Symptom Validity Test (MSVT) and Non-Verbal MSVT (NV-MSVT), the Coding (CD) and Digit Span (DS) subtests of the Wechsler Adult Intelligence Scale–Revised (WAIS-R), the Copy Trial of the Rey Complex Figure Test (RCFT_*Copy*_), the Wisconsin Card Sorting Test (WCST), and the California Verbal Learning Test (CVLT). Exclusion criteria were a diagnosis of intellectual disability or dementia. None of the patients included in this study were used in previous research on calibrating the EI model.

Mean age in the sample was 45.9 years (*SD* = 11.2), while mean level of education was 13.0 years (*SD* = 2.5). Mean self-reported depression was in the mildly elevated range, whereas anxiety was in the non-clinical range. The most common diagnosis was traumatic brain injury (TBI; 22.8%), followed by a variety of psychiatric (21.5%) and neurological (17.5%) disorders. The majority of TBI cases were of mild severity (*m*TBI; 70.0%). Most referrals were for independent medical examination (IME; 45.8%), followed by clinical evaluations (30.2%), medico-legal assessments in the context of litigation (M-LEG; 9.5%), and workplace-related injuries seeking compensation (W-COM; 8.8%).

### Materials

The WMT (Green, 2003) is presented to the examinee as a measure of verbal memory that consists of two learning trials, during which a list of words is displayed visually. Memory of the target words is assessed using a combination of cued paired associates recall, recognition, and free recall paradigms. The MSVT (Green, 2004) is similar to the WMT in format, but it is shorter: It contains half the target words (different stimulus material) and fewer time delays. Although it shares several features with the WMT (word pairs presented twice during the learning trials, forced-choice recognition [FCR] paradigm, computer administration), the MSVT is a well-validated free-standing PVT in its own right with strong empirical support (Armistead-Jehle & Denney, 2015; Armistead-Jehle et al., 2021; Carone, 2008, 2014; Green & Flaro, 2015; Kirkwood et al., 2012; Larson et al., 2015; Macciocchi et al., 2017). The NV-MSVT (Green, 2008) is a computer-administered free-standing PVT that is based on pictorial stimuli using the FCR paradigm. The utility of the NV-MSVT in differentiating between credible and non-credible neurocognitive profiles is well-established (Armistead-Jehle & Denney, 2015; Armistead-Jehle & Gervais, 2011; Armistead-Jehle & Hansen, 2011; Armistead-Jehle et al., 2021; Green, 2011; Green & Flaro, 2021; Henry et al., 2010). The TOMM-1 was also administered to 239 patients. Although originally developed as an inactive learning trial (Tombaugh, 1996), the TOMM-1 has evolved into a single-trial free-standing PVT in its own right (Denning, 2012) with a growing empirical evidence base (Denning, 2014, 2021; Fazio et al., 2017; Hilsabeck et al., 2011; Jones, 2013; Kulas et al., 2014; Martin et al., 2020; O’Bryant et al., 2003; Rai & Erdodi, 2021).

The WAIS-R versions of the CD and DS subtests were administered to all patients to preserve the consistency of the neuropsychological battery over time. Validity cutoffs for these measures were first developed on the WAIS-R version (Greiffenstein et al., 1994; Trueblood, 1994) and later replicated on subsequent editions of the CD (Ashendorf et al., 2017; Erdodi & Abeare, 2020; Erdodi, Abeare, et al., 2017; Erdodi & Lichtenstein, 2017; Etherton et al., 2006; Inman & Berry, 2002; Kim et al., 2010) and DS (Babikian et al., 2006; Erdodi & Abeare, 2020; Erdodi & Lichtenstein, 2017; Heinly et al., 2005; Mathias et al., 2002; Pearson, 2009; Schroeder et al., 2012; Shura et al., 2020; Whitney et al., 2009). The physical format of the stimulus material for the CD subtest became larger during each revision (i.e., WAIS-R < WAIS-III < WAIS-IV), resulting in a predictable shift in the raw score to scaled score conversion. However, as validity cutoffs were typically in age-corrected scaled scores (ACSSs), and this metric represents *relative standing* (i.e., performance compared with the rest of the normative sample within the same age group based on the same stimulus material) rather than absolute performance, they have the same interpretation across test versions. For example, an ACSS of 4 indicates that an examinee scored in the bottom 2% of the distribution regardless of the actual raw score, which may differ across editions. Therefore, the predictive power of validity cutoffs can be reasonably expected to transfer from one edition to the next.

In contrast, the Reliable Digit Span (RDS) is based on raw scores. Although the administration protocol is similar across WAIS-R and WAIS-III, the DS subtest underwent significant changes during the latest edition (i.e., WAIS-IV). In addition to introducing a third task (Digit Sequencing), the number of opening trials (i.e., digit span of two) was doubled (from two to four) on Digits Backward. Theoretically, this structural change to the test could have resulted in an increase in RDS scores on the WAIS-IV version, as examinees were provided with an extra opportunity to establish RDS backward of two. However, in practice, clearing this performance threshold is rarely a challenge. In a large clinical sample (*n* = 1,407), all of the patients with moderate/severe TBI obtained an RDS backward of ≥2 (Heinly et al., 2005). Likewise, Young et al. (2012) reported that even among patients who failed the WMT, the mean RDS backward was 3.1 (*SD* = 1.0). Similarly, Reese et al. (2012) found that mean RDS backward was 3.5 (*SD* = 1.1) among participants with mild head injury, and 2.7 (*SD* = 1.1) among experimental malingerers. Finally, a meta-analytic review by Jasinski et al. (2011) provided a direct comparison of the utility of WAIS-R and WAIS-III versions of DS as a PVT, and found essentially identical effect sizes (Cohen’s *d* of 1.26 and 1.28, respectively). Overall, these findings suggest that RDS scores from different versions of the test have comparable psychometric properties.

Given consistent recommendations that the final decision about performance validity should be based on multiple instruments (Boone, 2007; Larrabee, 2012a; Slick et al., 1999), the WMT, MSVT, and NV-MSVT were aggregated into a single metric (G-3). Passing all three at standard cutoffs (*n* = 300 or 66.4%) was considered an overall *Pass*. Patients who only failed one of the three PVTs (*n* = 71 or 15.7%) were excluded from the G-3 as a dichotomous variable, as a single PVT failure is considered insufficient to render the entire profile *invalid* (Boone, 2013; Larrabee, 2014a, 2014b; Schroeder et al., 2021). Consistent with established practice (Larrabee, 2012a; Victor et al., 2009), failing at least two of the three PVTs was considered an overall *Fail*. A five-variable version of the Erdodi Index (EI-5) was developed based on the following EVIs: CD, RCFT_*Copy*_, failures to maintain set on the WCST, RDS, and the Yes/No recognition hits raw score from the CVLT.

### Procedure

Data were collected through a retrospective archival chart review. The clinical files were irreversibly de-identified: All personal patient information was removed prior to capturing the data for research. The project was approved by the university Research Ethics Board. Ethical guidelines regulating research with human participants were followed throughout the study.

### Data Analysis

Descriptive statistics (mean, standard deviation [*SD*], BR_*Fail*_) were reported for key variables. Inferential statistics included independent *t* tests, one-way ANOVAs, and the χ^2^ test of independence. Effect size estimates were expressed in Cohen’s *d*, partial eta-squared $\left(\eta_{p}^{2}\right)$ and phi-squared (Φ^2^). Area under the curve (AUC) and the associated 95% confidence interval (CI) were computed in SPSS version 25.0. Likelihood ratios (LRs), sensitivity, specificity, and overall correct classification (OCC; the sum of true positives and true negatives divided by sample size) were calculated using standard formulas (Grimes & Schulz, 2005). The minimal acceptable level of specificity is .84 (Larrabee, 2003), although higher values (≥.90) are the emerging norm (Boone, 2013; Donders & Hayden, 2020).

BR_*Fail*_ is the proportion of individuals (%) in a sample who failed a PVT at a given cutoff. As such, BR_*Fail*_ is the sum of true positives (i.e., the proportion of non-credible patients who failed the PVT) and false positives (i.e., the proportion of credible patients who failed the PVT) divided by the sample size (*N*). The ratio of true positives (sensitivity) to true negatives (specificity) is typically determined based on the outcome of (an) external criterion measure(s). Although BR_*Fail*_ is not synonymous with false positive errors, BR_*Fail*_ provides the upper limit for the false positive rate (i.e., an individual who *passes* a PVT is at zero risk for failing it for reasons other than non-credible responding).

## Results

### Constructing the EI-5

As a first step, each component of the EI-5 is recoded onto a 4-point ordinal scale, ranging from 0 to 3 (i.e., levels of failure). Zero represents a score that passed the most liberal cutoff available. Therefore, it is considered an unequivocal *Pass* (i.e., it contains little to no evidence of non-credible responding). The first level of failure (EI component code of 1) is defined by the most liberal cutoff available in the literature or identified empirically (the first cutoff with at least .84 specificity). When the two sources of information conflict, the more conservative cutoff is applied. The second level of failure (EI component code of 2) is defined either by the next more conservative cutoff identified by previous research or the first cutoff that approximates a BR_*Fail*_ of 10% (for continuous scales that contain a wide range of potential cutoffs, such as the RCFT_*Copy*_). Finally, the third level of failure (EI component code of 3) represents either a previously calibrated ultra-conservative cutoff or the first cutoff that approximates a BR_*Fail*_ of 5% (Table 1).

**Table 1.** Base Rates of Failure across a Range of Cutoffs on the Components of the EI-5.

EI-5		Levels of failure				Criterion	
		**0**	**1**	**2**	**3**	G-3	
Component	Statistics	Pass	Fail	FAIL	**FAIL**	AUC	95% CI
CD_*WAIS-R*_		>6	6	5	≤4	.74	[.68, .79]
	BR_*Fail*_	88.1%	8.0%	3.1%	0.9%		
	SENS		.31	.12	.04		
	SPEC		.93	.97	1.00		
	OCC		.795	.793	.793		
							
Copy_*RCFT*_		>29.0	25.5–**29.0**	22.5–**25.0**	≤22.0	.73	[.66, .79]
	BR_*Fail*_	74.3%	15.0%	5.5%	5.1%		
	SENS		.53	.30	.15		
	SPEC		.84	.96	.99		
	OCC		.777	.816	.808		
							
FMS_*WCST*_		<3	3	4	≥5	.58	[.51, .66]
	BR_*Fail*_	84.7%	7.7%	4.2%	3.3%		
	SENS		.28	.15	.09		
	SPEC		.89	.95	.99		
	OCC		.761	.780	.798		
							
RDS		>7	7	6	≤5	.76	[.70, .84]
	BR_*Fail*_	83.6%	11.7%	3.8%	0.9%		
	SENS		.38	.12	.04		
	SPEC		.91	.98	1.00		
	OCC		.795	.801	.795		
							
RH_*CVLT*_		>12	12	11	≤10	.78	[.71, .84]
	BR_*Fail*_	85.2%	5.8%	3.3%	5.8%		
	SENS		.41	.30	.21		
	SPEC		.93	.97	.99		
	OCC		.822	.829	.827		

*Note.* Capitalization, boldface, and shading reflect increasing likelihood of non-credible performance; EI-5 = Erdodi Index Five; G-3 = The Green family of PVTs (Word Memory Test, Medical Symptom Validity Test, and Non-Verbal Medical Symptom Validity Test); AUC = area under the curve; CI = confidence interval; CD_*WAIS-R*_ = Digit-Symbol Coding subtest of the Wechsler Adult Intelligence Scale–Revised age-corrected scaled score (Ashendorf et al., 2017; Erdodi & Abeare, 2020; Erdodi, Abeare, et al., 2017; Etherton et al., 2006; Inman & Berry, 2002; Kim et al., 2010; Trueblood, 1994); BR_*Fail*_ = base rate of failure (percent of the sample that failed a given cutoff); SENS = sensitivity; SPEC = specificity; OCC = overall correct classification; Copy_*RCFT*_ = The Copy Trial of the Rey Complex Figure Test raw score (Abeare, An, et al., 2021; Lu et al., 2003; Rai et al., 2019; Reedy et al., 2013; Sugarman et al., 2016); FMS_*WCST*_ = Failures to Maintain Set on the Wisconsin Card Sorting Test raw score (DenBoer & Hall, 2007; Erdodi, 2021; Gligorović & Buha, 2013; Greve, Heinly, et al., 2009; Jodzio & Biechowska, 2010; Suhr & Boyer, 1999); RDS = Reliable Digit Span raw score (Babikian et al., 2006; Greiffenstein et al., 1994; Heinly et al., 2005; Mathias et al., 2002; Pearson, 2009; Schroeder et al., 2012; Shura et al., 2020; Whitney et al., 2009); RH_*CVLT*_ = Yes/No Recognition Hits (true positives), the California Verbal Learning Test raw score (Bauer et al., 2005; Curtis et al., 2006; Greve, Curtis, et al., 2009; Sweet et al., 2000; Trueblood, 1994). For Copy_*RCFT*_, where EI-levels are defined by a range of values, the cutoff used to compute classification accuracy is marked in boldface; *Pass* defined as passing all three at standard cutoffs; *Fail* defined as failing ≥2 at standard cutoffs).

The classification accuracy of individual components of the EI-5 was computed against the G-3 as criterion PVT. All five EVIs produced a significant AUC (range = .58–.78). The cutoffs defining the first level of failure on the EI-5 produced combinations of sensitivity (.28–.53) and specificity (.84–.93) hovering around the *Larrabee limit* (i.e., .50 sensitivity at .90 specificity; Crişan et al., 2021). Cutoffs corresponding to the second level of failure on the EI-5 disproportionately sacrificed sensitivity (.12–.32) for specificity (.95–.98). Finally, cutoffs corresponding to the third level of failure on the EI-5 produced perfect specificity (.99–1.00) but very low sensitivity (.04–.21).

The value of the EI-5 scale is obtained by summing its recoded components. As such, the scale ranges from 0 (*all five EVIs were passed at the most liberal cutoff*) to 15 (*all five EVIs were failed at the most conservative cutoff*). An EI-5 value ≤1 is considered a *Pass*, as it contains at most one marginal PVT failure. The interpretation of the next two levels (2 and 3) is more challenging, as they could reflect up to three marginal failures or one soft *Fail* and one hard *Fail*. Neither combination provides sufficiently strong evidence to deem the entire profile invalid. At the same time, EI-5 values two and three contain too much evidence of non-credible responding to classify them as valid. Therefore, this range is labeled *Borderline*, and omitted from measurement models that require a binary (*Pass/Fail*) outcome. In contrast, EI-5 values ≥4 indicate either at least four marginal PVT failures, at least two failures at the conservative cutoff or some combination of both. As such, this multivariate cutoff is considered the demarcation line for the *Fail* range (Erdodi, 2019, 2021).

The modal value on the EI-5 was zero (46.6%), indicating that almost half of the sample did not come close to failing any of the component EVIs. Two thirds of the sample (66.2%) scored in the *Pass* range (≤1), whereas one fifth (21.7%) scored in the *Borderline* range. Consequently, 12.2% scored in the *Fail* range (≥4). Of these patients, the majority (8.0%) scored 4 or 5, whereas 4.2% scored ≥6. A small proportion of patients (1.7%) scored ≥8.

### Classification Accuracy of the EI-5 Against Criterion PVTs

The EI-5 was a significant predictor of all four criterion PVTs (AUC = .77–.86). An EI-5 cutoff of ≥1 was highly sensitive (.81–.90), but insufficiently specific (.54–.59). OCC ranged from 60.2% to 65.9%. Raising the cutoff to ≥2 produced a sensible trade-off between improved specificity (.75–.81), diminishing sensitivity (.66–.78), and a notable increase in OCC (74.1%–80.1%). Making the cutoff more conservative (≥3) cleared the minimum specificity standard (.85–.93), while maintaining high levels of sensitivity (.61–.74) and OCC (82.3%–89.1%). The standard cutoff (≥4) further consolidated specificity (.92–.97) and OCC (84.7%–91.7%) at a proportional cost to sensitivity (.51–.65). This trend (slowly improving specificity and OCC in the backdrop of steadily declining sensitivity) continued at more conservative cutoffs (Table 2).

**Table 2.** Classification Accuracy of the EI-5 and TOMM-1 Against Criterion PVTs (n = 452).

EI-5	BR_*Fail*_	Criterion PVT											
		WMT			MSVT			NV-MSVT			G-3		
		24.3			18.6			17.3			17.9		
		AUC = .77 [.72, .83]			AUC = .81 [.75, .86]			AUC = .80 [.74, .86]			AUC = .86 [.81, .91]		
		SENS	SPEC	OCC	SENS	SPEC	OCC	SENS	SPEC	OCC	SENS	SPEC	OCC
≥1	53.5	.81	.55	.615	.87	.54	.602	.89	.54	.602	.90	.59	.659
≥2	33.8	.66	.77	.741	.74	.75	.750	.74	.75	.746	.78	.81	.801
≥3	20.1	.61	.89	.823	.69	.87	.838	.68	.85	.826	.74	.93	.891
≥4	12.2	.51	.94	.847	.59	.92	.873	.62	.92	.879	.65	.97	.917
≥5	6.4	.40	.98	.875	.48	.97	.905	.46	.96	.902	.54	.99	.926
≥6	4.2	.32	.99	.877	.37	.98	.912	.38	.98	.915	.44	1.00	.931
≥7	2.7	.23	1.00	.878	.29	.99	.920	.33	.99	.929	.36	1.00	.933
TOMM	BR_*Fail*_	Criterion PVT											
		WMT			MSVT			NV-MSVT			G-3		
		29.3			20.5			17.6			24.5		
		AUC = .83 [.76, .89)			AUC = .87 [.80, .93]			AUC = .80 [.71, .88]			AUC = .89 [.83, .95]		
		SENS	SPEC	OCC	SENS	SPEC	OCC	SENS	SPEC	OCC	SENS	SPEC	OCC
≤45	27.2	.63	.88	.803	.76	.85	.833	.69	.82	.795	.78	.90	.870
≤44	22.2	.54	.91	.803	.67	.90	.849	.60	.86	.812	.69	.92	.865
≤43	20.9	.53	.92	.808	.65	.91	.854	.60	.87	.824	.67	.93	.870
≤42	17.2	.49	.96	.820	.61	.94	.874	.57	.91	.854	.63	.97	.890
≤41	15.5	.44	.96	.812	.57	.95	.874	.55	.93	.862	.59	.98	.885
≤40	13.4	.39	.97	.799	.51	.96	.870	.48	.94	.858	.51	.99	.870
≤39	11.3	.34	.98	.795	.47	.98	.874	.45	.96	.870	.47	1.00	.870

*Note.* EI-5 = Erdodi Index Five; TOMM = Trial 1 of the Test of Memory Malingering; PVT = Performance Validity Test; BR_*Fail*_ = base rate of failure (percent of the sample that failed a given cutoff); WMT = Word Memory Test (standard cutoffs); MSVT = Medical Symptom Validity Test (standard cutoffs); NV-MSVT = Non-Verbal Medical Symptom Validity Test (standard cutoffs); G-3 = Number of failures on the Green family of PVTs (WMT, MSVT, and NV-MSVT at standard cutoffs; *Pass* defined as zero failures; *Fail* defined as ≥2 failures); AUC = area under the curve (95% confidence interval); SENS = sensitivity; SPEC = specificity; OCC = overall correct classification (the sum of true positives and true negatives divided by sample size).

### Classification Accuracy of the TOMM-1 Against Criterion PVTs

To provide a direct comparison between the EI-5 and a free-standing PVT, the calculations above were repeated with the TOMM-1 as the predictor. All AUC values were significant (.80–.89). The most liberal cutoff (≤45) cleared the minimum specificity threshold (.85–.90) against all criterion PVTs except the NV-MSVT (.82), at high sensitivity (.63–.78) and OCC (80.3%–87.0%). Lowering the cutoff to ≤44 shifted the signal detection profile in the predictable direction: improved specificity (.86–.92) and declining sensitivity (.54–.69), at comparable OCC (80.3%–86.5%). Making the cutoff more conservative (≤43) resulted in small increases in specificity (.87–.93), OCC (80.8%–87.0%), and shrinkage in sensitivity (.53–.67). The next level of cutoff (≤42) achieved uniformly high specificity (.91–.97) while maintaining sensitivity (.49–.63) and improving OCC (82.0%–89.0%). This trend of slowly improving specificity and deteriorating sensitivity in the context of slight fluctuations in OCC continued at more conservative cutoffs (Table 2).

### Five Shades of Gray: The Incremental Validity of the EI-5

**Figure 1.**
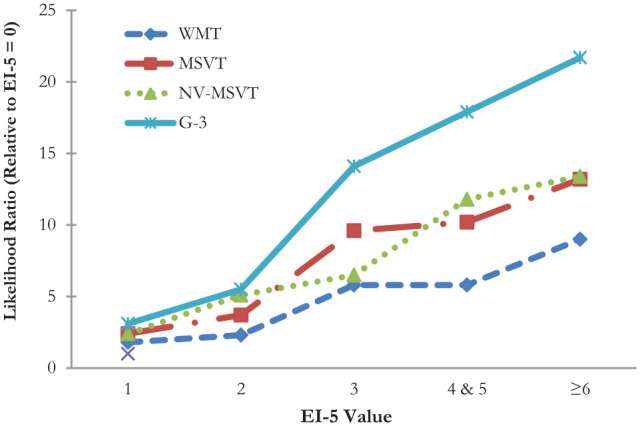
Changes in Likelihood Ratio Across EI-5 Values Relative to Base Rate of Failure at EI-5 = 0. *Note.* EI-5 = Erdodi Index Five; WMT = Word Memory Test (standard cutoffs); MSVT = Medical Symptom Validity Test (standard cutoffs); NV-MSVT = Non-Verbal Medical Symptom Validity Test (standard cutoffs); G-3 = Number of failures on the Green family of PVTs (WMT, MSVT, and NV-MSVT at standard cutoffs; Pass defined as zero failures; Fail defined as ≥2 failures).

At the EI-5 value of 0, BR_*Fail*_ on the free-standing PVTs was low (4.3%–10.0%). Patients with EI-5 scores of 1 (LR = 1.8–3.1) and 2 (LR = 2.3–5.4) were more likely to fail criterion PVTs. LRs were even higher at an EI-5 score of 3 (5.8–14.1). The trend of steadily growing LRs continued as EI-5 values increased. Figure 1 provides a visual summary of the linear relationship between the EI-5 and BR_*Fail*_ on the free-standing PVTs. The *y*-axis is skewed by the very large value associated with EI-5 ≥6 against the G-3 (LR = 21.7), suppressing the most important finding: LRs roughly doubled at each incremental increase in EI-5 values from 0 to 3.

### Between *Pass* and *Fail*: The Indeterminate Range

To evaluate the legitimacy of a three-way classification system, scores on free-standing PVTs as continuous variables were compared across the trichotomized EI-5 (*Pass*, *Borderline*, and *Fail*). The WMT, NV-MSVT, and MSVT produced extremely large main effects ( $\eta_{p}^{2}$ = .194–.242). All pairwise post hoc contrasts were significant. The largest effects were observed between *Pass* and *Fail* (*d* = 1.12–1.47; large), followed by the contrasts between *Borderline* and *Fail* (*d* = 0.62–0.74, medium-large). The smallest effects were observed between *Pass* and *Borderline* (*d* = 0.56–0.70; medium-large). A large main effect emerged on the TOMM-1 ( $\eta_{p}^{2}$ = .135). The *Pass* group scored significantly higher than both *Borderline* and *Fail* (*d* = 0.68–0.83; large). The contrast between *Borderline* and *Fail* did not reach significance. The *Pass* range of the EI-5 consistently produced the lowest level of within-group variability (Table 3).

**Table 3.** Scores on PVTs as Continuous Variables Across Ranges of EI-5 and the WMT.

PVT	EI-5 classification range						*F*	*p*	$\eta_{p}^{2}$	Sig. post hocs	*d*	σ_1_ vs. σ_2_
	Pass		Borderline		Fail							
	*n* = 299		*n* = 98		*n* = 55							
	*M*	*SD*	*M*	*SD*	*M*	*SD*						
WMT-3	284.5	20.4	265.1	33.6	237.8	40.0	78.9	<.001	.260	P-B	0.70	<.001
										P-F	1.47	<.001
										B-F	0.74	.136
MSVT-3	293.0	20.6	277.5	32.7	252.4	46.7	54.0	<.001	.194	P-B	0.57	<.001
										P-F	1.12	<.001
										B-F	0.62	.002
NV-MSVT	97.1	3.8	93.7	7.7	86.6	11.3	71.7	<.001	.242	P-B	0.56	<.001
										P-F	1.25	<.001
										B-F	0.73	.001
TOMM-1	47.5	3.2	44.0	6.5	42.9	7.2	18.4	<.001	.135	P-B	0.68	<.001
										P-F	0.83	<.001
										B-F	—	.499
PVT	WMT classification range						*F*	*p*	$\eta_{p}^{2}$	Sig. post hocs	*d*	σ_1_ vs. σ_2_
	Pass		Borderline		Fail							
	*n* = 312		*n* = 28		*n* = 112							
	*M*	*SD*	*M*	*SD*	*M*	*SD*						
TOMM-1	48.1	2.3	47.3	3.0	41.4	6.7	64.0	<.001	.351	P-B	—	.121
										P-F	1.34	.002
										B-F	1.14	<.001
EI-5	0.8	1.1	1.8	1.7	3.0	2.5	79.4	<.001	.261	P-B	0.70	<.001
										P-F	1.14	<.001
										B-F	0.56	.023

*Note.* PVT = Performance Validity Test; EI-5 = Erdodi Index Five; WMT-3 = sum of the first three trials on the Word Memory Test (total % correct; range = 0–300); Sig. post hocs = significant (*p* < .05) uncorrected post hoc contrasts; MSVT-3 = sum of the first three trials on the Medical Symptom Validity Test (total % correct; range = 0–300); NV-MSVT = Criterion A1 on the Non-Verbal Medical Symptom Validity Test (% correct; range = 0–100); TOMM-1 = Trial 1 of the Test of Memory Malingering; σ_1_ versus σ_2_ = The *p*-value associated with the *F*-test comparing two standard deviations; P-B = *Pass* versus *Borderline*; P-F = *Pass* versus *Fail*; B-F = *Borderline* versus *Fail*; WMT three-way classification (Pass = all three main subtests [IR, DR, and CNS] >85.0%; Borderline = at least one of the three main subtests [Immediate Recognition (IR), Delayed Recognition (DR), Consistency (CNS)] equals 85.0% [none < 85.0%]; Fail = at least one of the three main subtests [IR, DR, and CNS] is <85.0%).

The analyses above were repeated with the dichotomized (*Pass*/*Fail*) version of the PVTs (Table 4). A strong linear relationship emerged for all contrasts (Φ^2^ = .124–.356; large-extremely large effects). Among patients who passed the EI-5 (≤1), BR_*Fail*_ ranged from 6.7% to 19.1%. A notably larger proportion of patients in the *Borderline* range of the EI-5 (2–3) failed the free-standing PVTs (26.5%–48.0%). Naturally, the highest BR_*Fail*_ emerged among those with EI-5 scores ≥4 (41.9%–87.3%). The *Borderline* range of the EI-5 was associated with higher likelihood of failure on the free-standing PVTs compared with *Pass* (LRs = 2.51–5.26). The change in likelihood of failure from *Borderline* to *Fail* was smaller (LRs = 1.43–2.28). Finally, failing the EI-5 (≥4) was associated with a much higher likelihood of failure (LRs = 4.57–12.0) on the free-standing PVTs compared with passing the EI-5 (≤1).

**Table 4.** Base Rate of Failure on Criterion PVTs Across the Classification Ranges on the EI-5.

PVTs	EI-5 classification range			χ^2^	*p*	Φ^2^	Pairwise contrasts		
	Pass	Borderline	Fail						
	(0–1)	(2–3)	(≥4)					LR	
	*n* = 299	*n* = 98	*n* = 55				B/P	F/B	F/P
WMT	12.4%	35.7%	69.1%	90.0	<.001	.199	2.88	1.94	5.57
MSVT	7.4%	30.6%	58.2%	91.3	<.001	.202	4.14	1.90	7.86
NV-MSVT	6.7%	26.5%	58.2%	93.8	<.001	.208	3.96	2.20	8.69
TOMM-1	7.3%	29.3%	41.9%	29.6	<.001	.124	4.01	1.43	5.74
G-3 ≥1	19.1%	48.0%	87.3%	108.3	<.001	.240	2.51	1.82	4.57
G-3 ≥2	6.9%	36.3%	82.9%	135.8	<.001	.356	5.26	2.28	12.0
					Mean LR		3.79	1.93	7.41

*Note.* PVT = Performance Validity Test; EI-5 = Erdodi Index Five; LR = likelihood ratio; B/P = *Borderline* over *Pass*; F/B = *Fail* over *Borderline*; F/P = *Fail* over *Pass*; WMT = Word Memory Test (standard cutoffs); MSVT = Medical Symptom Validity Test (standard cutoffs); NV-MSVT = Non-Verbal Medical Symptom Validity Test (standard cutoffs); TOMM-1 = Trial 1 of the Test of Memory Malingering (*Fail* defined as ≤42; Greve et al., 2006; Greve, Etherton, et al., 2009; Denning, 2012; Jones, 2013; Kulas et al., 2014; Martin et al., 2020; Rai & Erdodi, 2021; Schroeder et al., 2019; Soble et al., 2020); G-3 ≥1 = number of failures on the Green family of PVTs (WMT, MSVT, and NV-MSVT at standard cutoffs; *Pass* defined as zero failures; *Fail* defined as ≥1 failures); G-3 ≥2 = number of failures on the Green family of PVTs (WMT, MSVT, and NV-MSVT at standard cutoffs; *Pass* defined as zero failures; *Fail* defined as ≥2 failures).

Next, the EI-5 was compared with the TOMM-1 as continuous scale using a trichotomized WMT as criterion. *Pass* on the WMT was defined as a score >85.0% on all three of the main scales (Immediate Recognition (IR), Delayed Recognition (DR), Consistency (CNS)); *Borderline* was defined as at least one score of 85.0% (labeled as “Warning” by the technical manual, conceptually identical to the current term “indeterminate”; P. Green, personal communication) but none <85%; *Fail* was defined as at least one score <85.0% (i.e., standard cutoff). An extremely large main effect ( $\eta_{p}^{2}$ = .351) emerged on the TOMM-1. Two of the post hoc contrasts (*Pass* vs. *Fail* and *Borderline* vs. *Fail*) were also significant (*d* = 1.14–1.34; large effects). The *Fail* subsample had the highest level of within-group variability; there was no difference in the *SD*s of *Pass* versus *Borderline* groups. The EI-5 also produced an extremely large main effect ( $\eta_{p}^{2}$ = .261). All post hoc contrasts were significant for both the means (*d* = 0.56–1.15; medium-large effects) and *SD*s (Table 3).

### Relationship Between PVT Failures and Contextual Factors

Passing or failing the G-3 was unrelated to age and gender. However, patients who failed the G-3 had lower levels of education (*d* = 0.49; medium effect), obtained lower scores on tests of auditory verbal learning and concept formation (*d* = 1.04–1.33, large effects), reported higher levels of emotional distress and demonstrated a higher tendency toward symptom exaggeration (*d* = 0.40–0.55, medium effects). In addition, invalid performance was associated with higher within-group variability.

Patients who failed the EI-5 were older, had lower levels of education (*d* = 0.21–0.31; small effects), performed more poorly on tests of auditory verbal learning and concept formation (*d* = 1.38–1.49, large effects), and reported higher levels of emotional distress (*d* = 0.35–0.55, medium effects). Passing or failing the EI-5 was unrelated to gender and symptom exaggeration (Table 5). Failing the G-3 (but not the EI-5) was associated with significantly higher BR_*Fail*_ on a symptom validity scale (LRs = 2.85–3.28).

**Table 5.** Relationship Between PVT Outcome, Demographic, Cognitive, and Psychiatric Variables.

Variable	Criterion PVT: G-3				*t*/χ^2^	*p*	*d*	LR	σ_1_ vs. σ_2_
	Pass		Fail						
	*M*	*SD*	*M*	*SD*					
Age	44.7	11.6	47.8	9.9	1.84	.067	0.29	—	.107
Education	13.6	2.5	12.4	2.4	2.74	.006	0.49	—	.150
CVLT 1–5	53.5	9.6	39.3	12.0	9.65	<.001	1.31	—	.008
CVLT LD-FR	11.5	2.8	7.3	3.5	9.75	<.001	1.33	—	.030
WCST CAT	5.1	1.7	3.0	2.3	9.08	<.001	1.04	—	<.001
BDI-II	15.2	11.6	21.5	11.3	4.34	<.001	0.55	—	<.001
PAI-NIM	54.2	12.1	59.9	16.2	2.50	.013	0.40	—	.095
BR_*Fail*_ ≥ 73	7.4		21.1		6.73	.009	—	2.85	—
BR_*Fail*_ ≥ 81	3.2		10.5		4.06	.044	—	3.28	—
PAI-DEP	61.3	17.8	69.9	13.2	2.83	.005	0.55	—	.014
PAI-ANX	55.3	14.2	62.3	12.7	2.81	.005	0.52	—	.380
PAI-SOM	60.7	15.0	68.8	18.5	2.93	.004	0.48	—	.138
% Men	53.8		54.5		0.01	.924	—	1.01	—
Variable	Criterion PVT: EI-5				*t*/χ^2^	*p*	*d*	LR	σ_1_ vs. σ_2_
	Pass		Fail						
	*M*	*SD*	*M*	*SD*					
Age	45.0	11.6	48.4	10.3	2.35	.019	0.31	—	.106
Education	13.2	2.5	12.7	2.3	1.92	.013	0.21	—	.302
CVLT 1–5	53.5	9.8	39.1	11.1	11.5	<.001	1.38	—	.214
CVLT LD-FR	11.6	2.9	6.9	3.4	12.3	<.001	1.49	—	.043
WCST CAT	5.3	1.6	2.8	2.0	10.0	<.001	1.38	—	<.001
BDI-II	15.4	11.6	21.3	9.6	3.56	<.001	0.55	—	.082
PAI-NIM	54.8	12.0	56.9	16.8	0.85	.397	—	—	.074
BR_*Fail*_ ≥ 73	8.5		12.9		0.64	.426	—	1.52	—
BR_*Fail*_ ≥ 81	4.2		6.5		0.30	.583	—	1.55	—
PAI-DEP	61.5	17.6	66.6	14.3	1.53	.128	—		.064
PAI-ANX	54.6	13.5	59.1	12.1	1.77	.079	0.35	—	.327
PAI-SOM	61.3	15.4	67.5	13.9	2.11	.036	0.42	—	.288
% Men	56.0		54.3		0.07	.878	—	0.97	—

*Note.* PVT = Performance Validity Test; G-3 = Number of failures on the Green family of PVTs (WMT, MSVT, and NV-MSVT at standard cutoffs); LR = likelihood ratio; σ_1_ versus σ_2_ = the *p* value associated with Levene’s test of equality of variance; CVLT = California Verbal Learning Test; 1–5 = sum of acquisition trials (raw score); LD-FR = Long-Delay Free Recall (raw score); WCST CAT = categories completed on the Wisconsin Card Sorting Test (raw score); BDI-II = Beck Depression Inventory–Second Edition (raw score); PAI = Personality Assessment Inventory (T-score); NIM = Negative Impression Management; BR_*Fail*_ = base rate of failure (percent of the sample that failed a given cutoff); DEP = Depression; ANX = Anxiety; SOM = Somatic Concerns; *Pass* defined as zero failures; *Fail* defined as ≥2 failures); EI-5 = Erdodi Index Five; Sig. post hocs = significant (*p* < .05) post hoc contrasts.

Next, the relationship between referral source and BR_*Fail*_ was examined across PVTs (Table 6). On average, the highest BR_*Fail*_ were observed in W-COM patients (29.3%), followed by IME (23.8%), M-LEG (21.5%), and clinical assessments (16.1%). External incentives to appear impaired were inferred from referral sources: W-COM, M-LEG, and IME were considered positive; clinical referrals were considered negative. Patients with positive incentive status were on average 1.52 times more likely to fail PVTs. There was no difference in BR_*Fail*_ across the three groups with positive incentive status (*p* = .136–.819).

**Table 6.** The Relationship Between Referral Source and Base Rates of Failure Across PVTs.

PVTs	*n*	Referral source					χ^2^	*p*	Φ^2^
		W-COM	M-LEG	Clinical	IME	Total			
		39	50	134	203	426			
EI-5	336	21.4	14.3	11.0	19.1	16.1	3.84	.279	.011
WMT	426	30.8	22.0	18.7	27.6	24.4	4.53	.210	.011
MSVT	426	28.2	26.0	12.7	18.7	18.5	7.30	.063	.017
NV-MSVT	426	25.6	10.0	14.9	20.7	18.1	5.54	.136	.013
TOMM-1	218	21.9	23.1	13.0	20.0	19.7	0.93	.819	.004
G-3 ≥1	426	43.6	34.0	26.9	36.9	34.0	5.42	.144	.013
G-3 ≥2	358	33.3	21.4	15.5	23.4	21.5	5.54	.136	.015
Mean BR_*Fail*_		29.3	21.5	16.1	23.8	21.8	—	—	—
PVTs	*n*	External incentive status		426	χ^2^	*p*	Φ^2^	LR	
		Negative	Positive						
		134	292						
EI-5	336	11.0	18.5	16.1	3.07	.080	.009	1.68	
WMT	426	18.7	27.1	24.4	3.51	.061	.008	1.45	
MSVT	426	12.7	21.2	18.5	4.41	.035	.010	1.67	
NV-MSVT	426	14.9	19.5	18.1	1.31	.252	.003	1.31	
TOMM-1	218	13.0	20.5	19.7	0.73	.395	.003	1.58	
G-3 ≥1	426	26.9	37.3	34.0	4.48	.034	.103	1.39	
G-3 ≥2	358	15.5	24.0	21.5	3.65	.056	.010	1.57	
Mean BR_*Fail*_		16.1	23.7	21.8				1.52	

*Note.* PVT = Performance Validity Test; W-COM = Workplace Injury Compensation; M-LEG = Medicolegal; IME = Independent Medical Examination requested by insurance companies; EI-5 = Erdodi Index Five; TBI = traumatic brain injury; WMT = Word Memory Test (standard cutoffs); MSVT = Medical Symptom Validity Test (standard cutoffs); NV-MSVT = Non-Verbal Medical Symptom Validity Test (standard cutoffs); TOMM-1 = Trial 1 of the Test of Memory Malingering (*Fail* defined as ≤43; Greve et al., 2006, 2009; Denning, 2012; Jones, 2013; Kulas et al., 2014; Martin et al., 2020; Rai & Erdodi, 2019; Schroeder et al., 2019; Soble et al., 2020); G-3 ≥1 = number of failures on the Green family of PVTs (WMT, MSVT, and NV-MSVT at standard cutoffs; *Pass* defined as zero failures; *Fail* defined as ≥1 failures); G-3 ≥2 = Number of failures on the Green family of PVTs (WMT, MSVT, and NV-MSVT at standard cutoffs); BR_*Fail*_ = base rate of failure (percent of the sample that failed a given cutoff); *Pass* defined as zero failures; *Fail* defined as ≥2 failures); Negative incentive status = clinical referrals; Positive incentive status = W-COM, M-LEG, and IME.

To examine the incremental utility of EI-5 over the G-3, a series of ANOVAs were performed with the following groups as the independent variable: passed both; passed one, failed the other; and failed both. A significant, but small main effect emerged for age and education: Patients who passed both PVTs were younger and better educated. There was no difference on gender and incentive status (Table 7). Extremely large main effects emerged on the CVLT and WCST ( $\eta_{p}^{2}$ = .249–.293), with a strong linear relationship between PVT failure and performance on cognitive testing. Failing *either* versus *both* criterion measures was associated with large effects (*d* = 0.83–0.94). The largest main effect was observed on TOMM-1 as a continuous variable ( $\eta_{p}^{2}$ = .388). Only 7.5% of patients who passed both the G-3 and EI-5 failed the TOMM-1. Those who failed either of the criterion PVTs were almost six times more likely to fail the TOMM-1 (LR = 5.72); patients who failed both criterion PVTs were more than eight times more likely to fail the TOMM-1 (LR = 8.43). Failing both versus just one of the criterion PVTs was associated with a 1.5 times higher likelihood of failing the TOMM-1. The main effect on the TOMM-1 was 1.5 times larger than what was observed on the CVLT and WCST, indicating a differential sensitivity to scores on PVTs relative to tests of cognitive functioning.

**Table 7.** Demographic Variables and Neuropsychological Test Scores Across the Joint Classification of the G-3 and EI-5.

Variable	Joint outcome of G-3 and EI-5						*F*/χ^2^	*p*	$\eta_{p}^{2}$	Sig. post hocs	*d*
	Passed both^A^		Pass/Fail^B^		Failed both^C^						
	*M*	*SD*	*M*	*SD*	*M*	*SD*					
Age	44.3	11.7	49.5	9.5	47.5	10.6	3.23	.041	.021	A-B	0.49
Education	13.5	2.5	13.2	2.8	12.3	1.9	3.33	.037	.022	A-C	0.54
Men	54.5%		52.0%		58.8%		0.31	.857	—	—	—
+INC-stat	65.1%		70.8%		75.8%		1.68	.432	—	—	—
CVLT 1–5	54.5	9.6	45.4	9.4	35.5	11.5	61.6	<.001	.293	A-B	0.96
										A-C	1.79
										B-C	0.94
CVLT LD-FR	11.9	2.7	9.1	3.1	6.4	3.4	61.7	<.001	.293	A-B	0.96
										A-C	1.79
										B-C	0.83
WCST CAT	5.3	1.6	4.2	2.2	2.4	1.9	49.3	<.001	.249	A-B	0.57
										A-C	1.65
										B-C	0.88
TOMM-1	48.2	2.3	44.9	4.6	40.0	7.1	47.5	<.001	.388	A-B	0.91
										A-C	1.55
										B-C	0.82
BR_*Fail*_ ≤43	7.5%		42.9%		63.2%		41.7	<.001	—	—	—
BDI-II	14.5	11.3	19.4	8.7	21.8	9.1	8.09	<.001	.053	A-B	0.49
										A-C	0.71
PAI-NIM	54.1	11.8	58.9	8.7	58.5	20.7	1.71	.183	.018	None	—
PAI-DEP	60.2	17.6	75.7	14.1	67.0	12.6	6.16	.003	.062	A-B	0.97
PAI-ANX	54.1	13.6	60.8	8.4	61.3	14.0	3.55	.031	.037	A-C	0.52
PAI-SOM	59.9	15.0	73.2	14.6	70.5	14.2	8.30	<.001	.082	A-B	0.90
										A-C	0.73

*Note.* G-3 = number of failures on the Green family of PVTs (WMT, MSVT, and NV-MSVT at standard cutoffs; *Pass* defined as zero failures; *Fail* defined as ≥2 failures); EI-5 = Erdodi Index Five (*Fail* defined as ≥4); Pass/Fail^B^ = passed one, failed the other; +INC-stat = positive external incentive status (presence of identifiable source of motivation to appeared impaired); CVLT = California Verbal Learning Test; 1–5 = sum of acquisition trials (raw score); LD-FR = long-delay free recall (raw score); WCST CAT = categories completed on the Wisconsin Card Sorting Test (raw score); TOMM-1 = Trial 1 of the Test of Memory Malingering; BR_*Fail*_ ≤ 43 = base rate of failure at 43 (% of the sample that scored ≤43 on the TOMM-1); BDI-II = Beck Depression Inventory–Second Edition (raw score); PAI = Personality Assessment Inventory (T-score); NIM = Negative Impression Management; DEP = Depression; ANX = Anxiety; SOM = Somatic Concerns; Sig. post hocs = significant (*p* < .05) uncorrected post hoc contrasts; A, B, C = identify the pairwise contrasts (i.e., A-B, A-C, B-C).

However, symptom validity was unrelated to the joint outcome of the G-3 and EI-5 (Table 7). Patients who failed either the G-3 or EI-5 reported higher levels of depression than those who passed both (medium-large effect). Failing both criterion PVTs was associated with higher levels of anxiety than passing both (medium effect). Patients who failed either or both criterion PVTs reported higher levels of somatic symptoms (large effects). Compared with the contrasts based on the G-3 or EI-5 alone as grouping variables, effect sizes were larger between patients who passed both versus those who failed both criterion PVTs on measures of cognitive ability (*d* = 1.04–1.49 vs. 1.65–1.79) and self-reported emotional distress (*d* = 0.48–0.55 vs. 0.52–0.97).

Finally, to evaluate the link between genuine cognitive deficits and non-credible performance, BR_*Fail*_ were compared across injury severity within the TBI subsample (*n* = 103). Patients with mild TBI had higher BR_*Fail*_ on free-standing PVTs (LRs = 1.99–4.71), consistent with previous research (Erdodi & Rai, 2017; Heinly et al., 2005; Hurtubise et al., 2020). However, no differences were observed in BR_*Fail*_ on the EI-5 (LR = 1.04) as a function of injury severity.

## Discussion

This study was designed to cross-validate the EI, a novel method of aggregating EVIs against well-established and widely used free-standing PVTs (Martin et al., 2015; Sharland & Gfeller, 2007) in a large mixed clinical-forensic sample. Results converge in a number of main findings. At the standard cutoff (≥4), the EI-5 produced a good combination of sensitivity (.65) and specificity (.97), correctly classifying 92% of the sample. Its classification accuracy was comparable with the TOMM-1. A more liberal EI-5 cutoff (≥3) also achieved good classification accuracy (.74 sensitivity at .93 specificity and 89% OCC). Evidence suggests that the indeterminate (*Borderline*) range is a legitimate third outcome of performance validity testing that is psychometrically distinct from both *Pass* and *Fail*. Additional findings with methodological and clinical relevance are summarized below.

### E Pluribus Unum

As an aggregate measure of performance validity, the EI-5 had superior classification accuracy to its individual components, correctly classifying an additional 10% to 15% of the sample, consistent with previous research (Erdodi, 2021). Even though two of its components failed to reach .90 specificity at the most liberal cutoff, at ≥4, the composite was highly specific against both individual PVTs (.92–.94) and the G-3 (.97), suggesting that the whole is larger than the sum of its parts. Therefore, the signal detection profile of individual PVTs in a multivariate model is less relevant than the classification accuracy of the aggregate index. Once validity cutoffs are combined, their individual signal detection profiles become irrelevant, as they are transformed by the aggregate measure. As long as the multivariate cutoff is sufficiently specific, it does not matter if any of the individual components is associated with elevated false positive rate.

In fact, the EI model reveals an important shortcoming of traditional multivariate models of PVTs: Insisting that all individual components meet a high specificity standard (i.e., ≥.90) comes at a (perhaps unnecessarily) high cost to sensitivity. Therefore, the first level of failure on the EI is optimized for the detection of psychometrically ambiguous (“gray-zone”) manifestations of non-credible performance. Put differently, at the intake end, the model has a deliberate alpha-bias: It is designed to identify even mild forms of invalid responding. At the output end, however, the model has a beta-bias: the evidence is carefully weighted, and the multivariate cutoff is optimized for specificity. Consequently, it allows for up to three individual EVI scores in the *indeterminate* range before classifying the entire profile as *invalid*.

Essentially, the false positive rate of the composite is effectively controlled by applying a more stringent multivariate cutoff (Pearson, 2009). For example, an EI-5 cutoff of ≥1 would have an unacceptably high false positive rate (41%–46%). However, raising the cutoff to ≥3 keeps false positives low (7%–15%), and ≥4 further limits the false positive rate (3%–8%). In contrast, if a conservative cutoff were to be imposed on each component (i.e., EI level 2), as commonly practiced, the subthreshold level manifestations of non-credible responding (i.e., defined by EI level 1 cutoffs) would remain undetected, attenuating overall sensitivity.

In other words, insistence on conservative cutoffs at the level of individual PVTs forfeits the opportunity to identify an entire subtype of invalid performance: examinees who consistently perform at the outer edge of credible presentation, but without crossing overly cautious psychometric thresholds. Evidence suggests that such profiles are statistically uncommon (Pearson, 2009) and are strong predictors of psychometrically defined non-credible performance (Erdodi, 2019, 2021). However, this presentation is common in certain settings, such as university students volunteering for academic research (An et al., 2017; Roye et al., 2019) or baseline testing in sports concussion centers (Abeare et al., 2019).

### The EI-5 Versus the TOMM-1

Although EVIs are commonly considered inferior to free-standing PVTs (Martin et al., 2015; Shura et al., 2020), results do not support this claim. At their optimal cutoffs, the EI-5 and TOMM-1 produced comparable classification accuracy. This equivalence is remarkable given that the TOMM-1 had the modality specificity advantage (Lace et al., 2020; Rai & Erdodi, 2021; Schroeder et al., 2019), as it shares the FCR paradigm with the G-3, whereas 80% of the EI-5 components are based on non-FCR tests. This finding demonstrates that while individual EVIs may have substandard classification accuracy, when combined in a multivariate model, their cumulative signal detection performance is at par with free-standing PVTs (Erdodi, 2021).

### The Unique Contributions of the EI-5 to Performance Validity Assessment

The most compelling argument for using EVIs is an economic one: They represent a significant saving in test material and administration/scoring time. However, results also suggest that the EI model has incremental utility even when used in combination with several expansive, well-established free-standing PVTs, consistent with previous reports (Erdodi, et al., 2019). There was a strong linear relationship between the performance validity gradient (*Pass* both, *Fail* either, and *Fail* both) defined by the dual criteria (G-5 and EI-5) and scores on cognitive tests and the TOMM-1, suggesting that applying an EI model even on top of highly effective free-standing PVTs provides additional evidence on the credibility of neuropsychological profiles.

Specifically, patients who failed both the G-3 and the EI-5 were 1.5 times more likely to fail the TOMM-1 than those who only failed one of the criterion PVTs. In other words, complementing existing methods of performance validity assessment with the EI model has the potential to enhance overall signal detection accuracy. Conversely, if the administration of multiple free-standing PVTs is not feasible due to practical constraints (abbreviated batteries in clinical or research settings, reviewing the results of existing neuropsychological assessments lacking dedicated PVTs), the EI model offers an empirically supported alternative approach to evaluating the credibility of the presentation.

### The Dual Nature of Performance Validity: Continuous Versus Dichotomous

From its inception, the EI model compresses a continuous scale into a 4-point ordinal scale (0, 1, 2, and 3), recognizing the need to reduce a wide range of performance into a small number of clinically meaningful categories. In turn, the value of the EI is further collapsed into three categories: *Pass*, *Borderline*, and *Fail*. At the same time, the model acknowledges at every step of the way that the underlying construct is a continuous variable, and forcing it into discrete categories imposes an artificial structure on the data. Therefore, the predictive value of the EI model must be evaluated empirically.

Results indicate that patients with an EI-5 score ≤1 fail free-standing PVTs at a low rate (6.7%–19.1%); those with EI-5 scores of 2 or 3 produced a higher BR_*Fail*_ (26.5%–48%); even higher BR_*Fail*_ were observed among those with EI-5 scores ≥4 (41.2%–87.3%). In fact, relative to an EI-5 value of zero, BR_*Fail*_ on criterion PVTs roughly doubled with each unit increase at the next three levels of the EI-5. The incremental change in the likelihood of non-credible responding demonstrates that a single cutoff (i.e., *Pass/Fail*) anywhere on the scale produces contaminated criterion groups. Declaring EI-5 values 2 and 3 a *Pass* would include patients with significant objective evidence of invalid performance. Even though evidence suggests that the *Borderline* range is closer to *Fail* than *Pass*, lowering the EI-5 cutoff to ≥3 may increase the risk of false positives beyond what many assessors consider acceptable.

Given the primacy of specificity in performance validity assessment, the threshold for failure on PVTs is deliberately high. Consequently, passing these cutoffs cannot be used as evidence of credible performance (Chafetz, 2020). The sharp increase in BR_*Fail*_ on free-standing PVTs from the *Pass* to the *Borderline* range of the EI-5 is an objective reminder of this principle. Habitually differentiating between levels of PVT failures (i.e., shades of gray) could help resolve this uncertainty around the clinical interpretation of profiles with *some*, but *not enough* evidence of invalid performance. For example, an EI-5 value of 0 and 3 would both be classified as “did not fail.” However, results clearly show that these two scores have vastly different predictive power: Patients with an EI-5 value of 3 are 6 to 14 times more likely to fail free-standing PVTs compared with those with an EI-5 value of 0. Indeed, an EI-5 cutoff of ≥3 was sufficiently specific (.93) to invalid performance in this sample.

### “Indeterminate” as a Legitimate Third Outcome of Performance Validity Testing

This strong linear relationship between EI-5 scores and BR_*Fail*_ on criterion PVTs supports the three-way classification scheme. The idea of allowing an “in-between” category that represents neither valid nor unequivocally invalid performance is not new (Ashendorf, 2019; Bigler, 2015; Green, 2003; Rogers & Granacher, 2011), but it seems to be gaining widespread acceptance (Guilmette et al., 2020). Formally recognizing a *Borderline* range between *Pass* and *Fail* would not only increase the purity of criterion groups in research studies, but also alleviate pressure on assessors to characterize individual neurocognitive profiles as *either* credible or non-credible, ignoring disconfirming evidence.

### Within-Group Variability as a Marker of Non-Credible Presentation

Invalid performance was frequently associated with inflated *SD*s, consistent with previous reports (Abeare, Hurtubise, et al., 2021; An et al., 2019). This phenomenon can be attributed to divergent malingering agenda or strategies (Cottingham et al., 2014; Mittenberg et al., 2002), the natural variability in individuals’ ability to feign credible impairment (Erdodi, Kirsch, et al., 2014) or abnormal fluctuations in test taking effort (Bigler, 2012). Regardless of etiology, this well-replicated phenomenon suggests that psychometrically, credible presentation converges in a narrow range of responses, whereas non-credible presentation is characterized by a divergence in response patterns. This emergent marker of invalid performance may be useful in group-level analyses and provides additional empirical support for within-profile consistency as a template for evaluating the credibility of an individual’s presentation (Sherman et al., 2020).

### The Effect of Contextual Variables

Failing the EI-5 was associated with older age, lower levels of education, and elevated self-reported psychiatric symptoms. There were no gender effects. Similarly, the EI-5 was orthogonal to TBI severity, as previously reported (Abeare, Sabelli, et al., 2019; Donders et al., 2021; Erdodi & Abeare, 2020). Being insensitive to the deleterious effects of genuine neurological impairment is a quintessential feature of a good PVT (Larrabee, 2012b; Messa et al., 2021; Strauss et al., 2006). Although patients with external incentives to appear impaired had a higher BR_*Fail*_ on the EI-5 (LR = 1.68), the contrast was not significant, consistent with the ongoing controversy around the salience of motivational states in performance validity assessment (Boone, 2013; Hurtubise et al., 2020; Larrabee et al., 2007).

### Limitations

The sample was diagnostically heterogeneous and geographically restricted. As such, it is unclear whether the findings would generalize to different regions (Lichtenstein et al., 2019), clinical populations, and instruments. At the same time, the study is based on a large sample representing a mixture of clinical and forensic referrals. Patients were evaluated using a rare combination of three well-established stand-alone PVTs and five EVIs that were administered to every individual in the sample, which is a significant methodological improvement over previous studies on the EI model (Erdodi, 2019; Erdodi et al., 2016; Erdodi, Roth, et al., 2014). Future studies would benefit from replicating these results in patients with different diagnoses using EI models built from different EVIs.

## Conclusion

Results support the use of the EI model to evaluate the credibility of cognitive deficits in both clinical and forensic settings. At the standard cutoff (≥4), the EI-5 correctly classified 92% of the sample; its classification accuracy compared favorably with the TOMM-1, and it was robust to the effect of genuine neurological impairment. The *Borderline* emerged as a legitimate third outcome of PVTs that deserves further endorsement from professional organizations. As the EI model is not an instrument, but a *method* of aggregating PVTs into a composite measure of performance validity, ongoing cross-validation research is needed to establish the limits of its generalizability. The inherent flexibility of the EI model makes it a versatile tool: Any combination of EVIs or free-standing PVTs can be transformed into a single-number summary of the cumulative evidence regarding the credibility of a given neurocognitive profile. The EI model has the potential to become a useful method for combining data from multiple instruments in both research and clinical settings.
